# Highly reproducible alkali metal doping system for organic crystals through enhanced diffusion of alkali metal by secondary thermal activation

**DOI:** 10.1038/s41598-018-26048-6

**Published:** 2018-05-16

**Authors:** Jinho Lee, Chibeom Park, Intek Song, Jin Young Koo, Taekyung Yoon, Jun Sung Kim, Hee Cheul Choi

**Affiliations:** 1 Center for Artificial Low Dimensional Electronic Systems, Institute for Basic Science (IBS), 77 Cheongam-ro, Nam-Gu, Pohang, 37673 Korea; 2 Department of Chemistry, Pohang University of Science and Technology (POSTECH), 77 Cheongam-ro, Nam-Gu, Pohang, 37673 Korea; 3 Department of Physics, Pohang University of Science and Technology (POSTECH), 77 Cheongam-ro, Nam-Gu, Pohang, 37673 Korea

## Abstract

In this paper, we report an efficient alkali metal doping system for organic single crystals. Our system employs an enhanced diffusion method for the introduction of alkali metal into organic single crystals by controlling the sample temperature to induce secondary thermal activation. Using this system, we achieved intercalation of potassium into picene single crystals with closed packed crystal structures. Using optical microscopy and Raman spectroscopy, we confirmed that the resulting samples were uniformly doped and became K_2_picene single crystal, while only parts of the crystal are doped and transformed into K_2_picene without secondary thermal activation. Moreover, using a customized electrical measurement system, the insulator-to-semiconductor transition of picene single crystals upon doping was confirmed by *in situ* electrical conductivity and *ex situ* temperature-dependent resistivity measurements. X-ray diffraction studies showed that potassium atoms were intercalated between molecular layers of picene, and doped samples did not show any KH- nor KOH-related peaks, indicating that picene molecules are retained without structural decomposition. During recent decades, tremendous efforts have been exerted to develop high-performance organic semiconductors and superconductors, whereas as little attention has been devoted to doped organic crystals. Our method will enable efficient alkali metal doping of organic crystals and will be a resource for future systematic studies on the electrical property changes of these organic crystals upon doping.

## Introduction

Organic molecular systems have gained much attention in recent years as a potential core component for various electronic devices such as organic light emitting diodes (OLED)^1^, organic field-effect transistors (OFET)^2^, and organic solar cells (OSC)^3^. Due to their excellent physical flexibility, optical transparency, and suitability for chemical modification, these devices are considered as attractive alternatives to current silicon-based devices. Nonetheless, only a few types of organic molecules, such as charge transfer complexes^4^, and highly aromatic molecules, such as rubrene^5^, have been suggested for these purposes with the hope of obtaining high electrical properties. The organic molecular systems have been pursued in many forms including polycrystalline powder, thin film, and single crystals. Considering that most of the efforts have been focused on finding the molecules with suitable electric properties in chemically unmodified pure states with no remarkable success, it would be highly beneficial to search for candidate systems that would exhibit such properties upon slight and mild chemical treatments such as traditional doping with alkali metals that have been shown to work very well for inorganic systems. Unfortunately, no efficient doping method is currently available, especially for organic single crystals.

Despite the highly conjugated electronic systems present in many candidate molecules, one of the significant challenges for the applications of the carbon-based organic systems in electronic devices is their intrinsically poor electrical conductivity. Although the extensive π electron system of organic molecules can provide a decent amount of charge carriers, this amount is still too low compared to inorganic systems.

A number of strategies have been suggested to increase the electrical conductivity of organic molecular systems including the substitution of carbon with electron-rich elements such as sulfur or selenium^4^ or modification of molecular structure under high pressure^6^. Doping, another traditional method for increasing the conductivity of inorganic systems, has been regarded as one of the most effective ways for increasing the conductivity of organic molecular systems. Doping directly supplies a large amount of charge carriers from the dopants to the target material, and it readily results in metallic conductivity or even superconductivity in some extreme cases (graphite^7^, MoS_2_^8^).

Alkali metal or other metal-doped polyaromatic hydrocarbon (PAH) molecules are examples of these extreme cases wherein doping can induce superconductivity in organic molecules^9,10^. Previous studies have confirmed that alkali metal doping induces superconductivity by injecting a substantial amount of electrons into the target^11^, even though the exact origin of the superconductivity is still unclear. More importantly, this effect is quite difficult to achieve especially for organic single crystals mainly because the traditional alkali metal doping into organic molecular systems has targeted mostly polycrystalline or highly granular specimen that are relatively easy to dope but are not suitable for reliable and systematic doping studies. For example, alkali metal doping to granular C_60_ results in an anomalous fluctuation in the temperature-dependent electrical conductivity, and this “noise” hinders precise investigations^11,12^.

An ideal solution to resolve this problem would be to use organic single crystals as a target. Since single crystals usually have a much lower populations of grain boundaries and charge traps compared to polycrystalline granular powders, they are excellent specimens for studying the behaviors of organic molecular systems upon doping. Nonetheless, such studies using organic single crystals are quite rare mainly because of the lack of efficient and reliable alkali metal doping method applicable to organic single crystals. It should be noted that the traditional doping method that relies heavily only on the thermally activated vaporized alkali metals that has worked quite well for polycrystalline or highly granular systems is not suitable for a single-crystalline specimen. This is because the compact, almost defect-free single crystals with only a limited space have much higher barrier for the diffusion of the dopants into the crystal. The traditional method is designed to induce spontaneous diffusion of vaporized alkali metal from a stoichiometric mixture of alkali metal and target organic systems, mostly in powder form. In addition, this process is relatively fast in granular specimen because the grain boundaries and defects that are highly populated in the granular specimen lower the activation energy of the diffusion process^13^; however, this cannot be achieved for organic single crystals by simply increasing the source heating temperature because of another important obstacle to the study of organic single-crystal systems. That is, the weak intermolecular interaction among the PAH molecules makes it difficult to tightly bind the neighboring molecules, thus leading to the destruction of the whole crystal during the doping process at high temperature. For the same reason, doping by an implantation technique such as ion bombardment that is often used for robust inorganic crystals cannot be applied to soft PAH crystals. Therefore, a new doping method that can promote diffusion more efficiently should be developed for soft PAH single crystals.

Herein, we report an efficient alkali metal doping system that enables uniform and reproducible doping of potassium into organic single crystals including picene. Our strategy is to further activate the PAH crystals by supplying extra mild heat to the target specimen as a secondary thermal activation during the doping process. Supplying heat only to the dopant source, which has been conventionally used in previous studies^14^, cannot yield the full intercalation of the dopants due to the reasons mentioned above. By contrast, the additional supply of mild heat to the target crystal as a secondary thermal activation source, which is unique in our system, enables further thermal activation of adsorbed potassium atoms so that the dopants are provided with additional kinetic energy to enhance the diffusion into an organic single crystal while minimizing the structural damage during the doping process. In addition, our doping system is equipped with a real-time measurement system that can analyze *in situ* electrical conductivity changes to monitor the doping process in real time. We also performed Raman spectroscopy, X-ray diffraction, and *ex situ* resistivity-temperature measurements to check the quality and uniformity of the doped specimens. We found that our system could effectively dope picene single crystals by observing the phase transition of the doped samples and found the expansion of the crystal lattice without damage. To the best of our knowledge, our system is the first example demonstrating the doping of organic single crystals with alkali metals, even with *in situ* monitoring of their electrical property changes upon doping. We believe that our novel doping method may provide better opportunities for the discovery of new organic crystals that could be potentially developed into organic semiconductors or superconductors.

## Results

**Figure 1:**
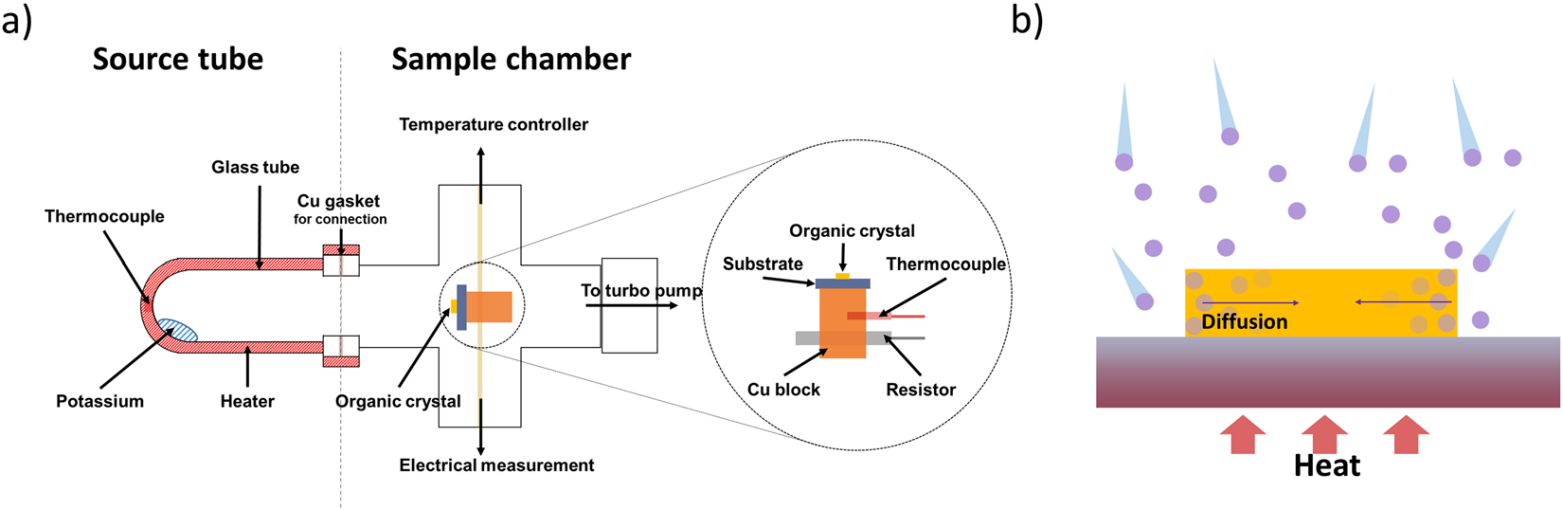
(**a**) Scheme of the enhanced diffusion method for doping the organic crystals with alkali metal. Inset shows expanded region of the heating block to control the temperature of specimens during the doping process. (**b**) Scheme of the process for alkali metal doped organic single crystal.

As seen in the schematic of the doping system shown in Fig. 1a, the doping system basically consists of two parts: the left part is the source chamber from which the potassium vapor is generated, and the right part is the sample chamber where the organic single-crystal devices are placed on top of the temperature-controllable stage. Maintenance of a uniform and proper temperature of the target crystal is the key factor for the decomposition-free, highly reproducible doping. To control the temperature of the target crystal on a SiO_2_ substrate, a resistor (as a heater) and a temperature sensor were connected to a Cu block attached to the substrate. The temperature of the source heater and the Cu block were respectively kept at 140 °C and 90 °C (Fig. 1a, inset) by turning the current to the resistor on and off. The evaporated potassium from the source chamber was then adsorbed onto the surface of the target crystal and diffused into the crystal with the help of secondary thermal activation.

### Growth and characterization of single crystals

**Figure 2:**
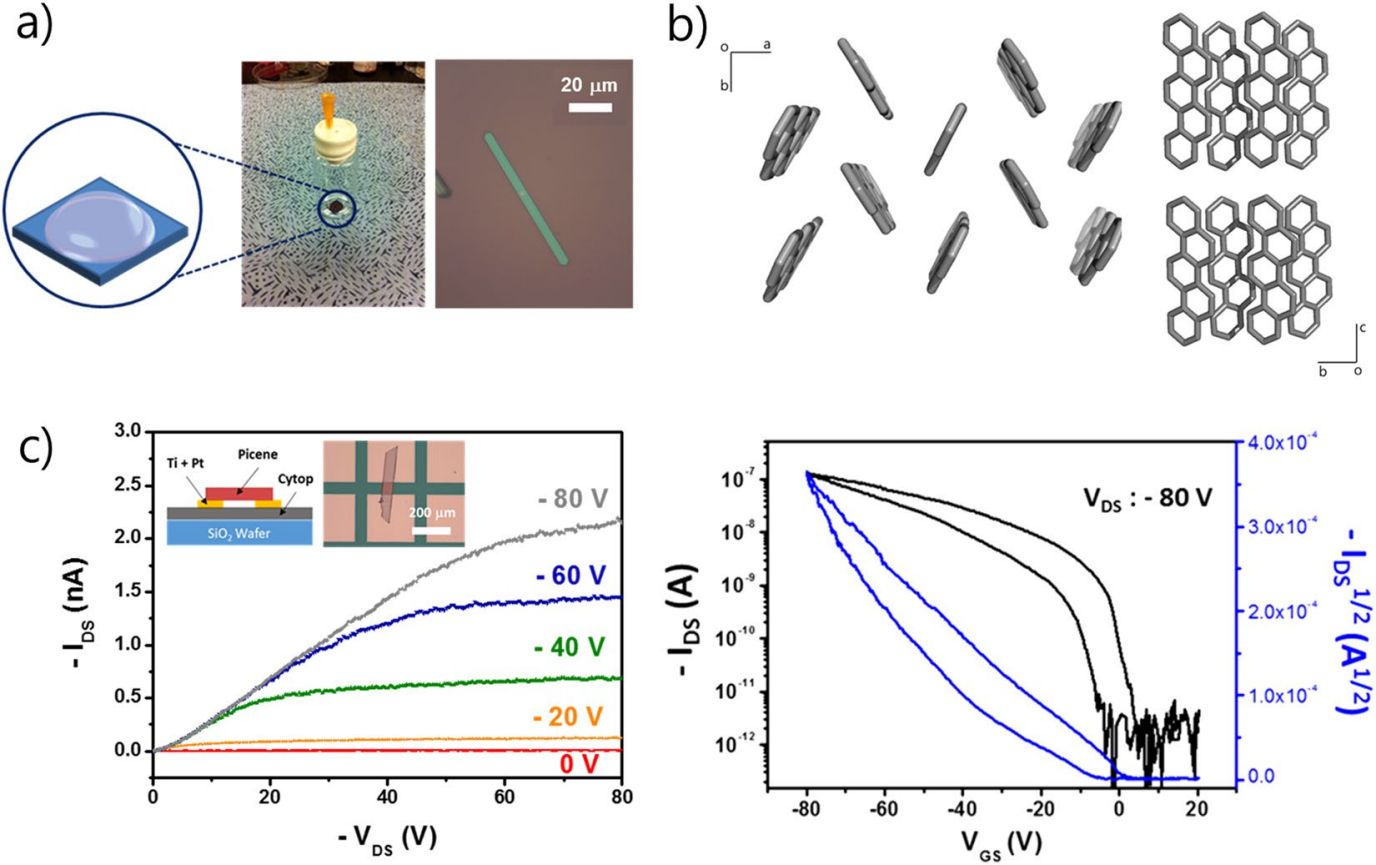
(**a**) Drop-drying method for growth of picene single crystal and optical microscopy image of picene single crystal. (**b**) Crystal structures of picene single crystal viewed from c-direction (left) and a-direction (right). (**c**) I_DS_-V_DS_ curves (left) and I_DS_-V_G_ curve (right) measured from pristine picene single crystal FET device. Insets schematically show device structure and optical microscopy image of the device.

We chose picene as the target molecule for the evaluation of the performance of the doping system. This representative PAH molecule has been reported to show superconductivity upon doping with alkali metals^9,10,15^. High-quality, belt-shaped picene crystals were prepared by a simple drop-drying method (Fig. 2a, left). (The details are explained in Methods). A representative optical image of the as-grown belt-type picene crystals is shown in Fig. 2a, right. The width of the crystal ranges from 5 μm to 50 μm with the length ranging from 100 μm to 500 μm. Scanning electron microscopy (SEM) and atomic force microscopy (AFM) studies show that the picene crystal has a flat, smooth surface, which is suitable for the fabrication of the bottom-electrode devices for the measurements of the electrical properties (Fig. S1). The crystal structure of the picene crystal (monoclinic, *P2*_1_, a = 8.4032(17) Å, b = 6.0742(12) Å, c = 13.457(3) Å, *β* = 90.06(3)°, *V* = 686 Å^3^) has a herringbone-like molecular arrangement (face-to-edge), with the herringbone layers (ab-plane) stacked along the c-axis (Fig. 2b), which well matches with the reported picene single crystal results^16^. It should be noted that although the crystallization occurs in solution, the crystal did not include any solvent molecules in the crystal structure, whereas C_60_ fullerene crystals do include the solvent molecules in the similar crystallization process^17^. We believe that this offers a clue as to the space available for the alkali metal intercalation.

### Measurements of electrical property changes of picene single-crystal upon doping

To examine the electrical properties of the as-prepared picene single crystals, we measured the electrical conductivity and the carrier mobility of the as-prepared picene single-crystal using the fabricated devices as described in methods, and in the inset of Fig. 2c. The electrical conductivity of the picene single-crystal was measured by two-probe I_DS_-V_DS_ measurements in vacuum at various temperatures (Fig. S2). The measured conductivity was 2.92 × 10^−8^ S/cm at room temperature. The temperature-dependent conductivity measurements show that the crystal is semiconducting. The V_G_-dependent I_DS_ – V_DS_ curves show a typical p-type semiconducting behavior (Fig. 2c). It can be seen from the I_DS_ – V_G_ curve measured in vacuum at room temperature that the carrier mobility of the picene single-crystal was calculated to be 4.73 × 10^−3^ cm^2^/V·s. This value is slightly lower than the values obtained from field-effect transistors with optimized configuration such as top-electrodes or optimized chemical passivation between the crystal and electrodes^18^. Nevertheless, the use of a pristine material with chemically pure electrodes is most effective for exploring the intrinsic properties of the channel material (picene in this case) and for the further doping experiments.

#### *In situ* measurements of electrical current during doping of picene single crystal

**Figure 3:**
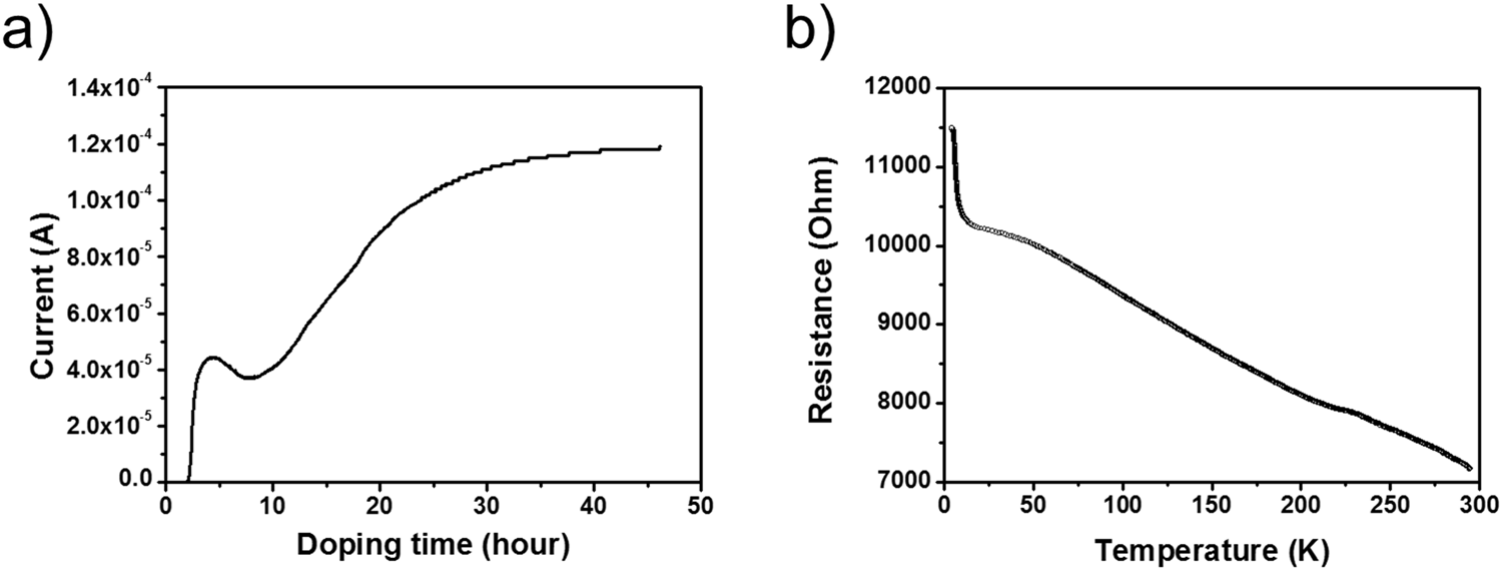
(**a**) Change of current during the alkali metal doping into picene single crystal at 90 °C. (**b**) Temperature dependence of electrical resistance in the potassium doped picene single crystal.

To characterize the changes of electrical properties by the doping process, we conducted *in situ* measurements for the electrical current change during the doping, as well as *ex situ* measurements for the temperature-dependent resistivity. The former measurements showed a dramatic increase of the electrical conductivity upon doping (Fig. 3a). Two hours of doping resulted in an increase of the current at first, followed by a small decrease. However, further doping once again increased the current which eventually saturated after 40 hours. The conductivity after 45 hours was 4.22 × 10^−1^ S/cm at room temperature, which is seven orders of magnitude higher than that of the pristine picene. This increase of the conductivity was reproduced more than ten times. Additionally, the sample showed Ohmic contact characteristics at low voltage regime. We note that when the measurements were performed for a set of four channels across the doped crystal, all of the measurements showed the same behavior (Fig. S3). This finding confirms the uniformity of the doped phase in the entire crystal. The increase in the conductivity originated from the partially filled energy bands derived from the π orbitals of picene similar to the case of the potassium-doped C_60_^19^. We note that such an increase of the conductivity is not attributed to the formation of a potassium layer on the substrate, because we confirmed the absence of such conductivity changes for the measurements on the bare substrate (Fig. S4). Based on the Raman spectrum and conductivity measurements, we concluded that the sample is doped to become K_2_picene rather than K_3_picene which is known to exhibit a superconducting phase^20^.

#### *Ex situ* measurements of temperature-dependent resistivity for the potassium-doped picene single crystal

We further examined the nature of the doped crystal with the measurements of the resistivity-temperature relationship. Direct current (dc) electrical resistivity was measured as a function of temperature for a K_2_picene single crystal. Using a four-probe measurement, the resistivity of the doped crystal was estimated to be 2.37 Ω⋅cm at room temperature. The resistance steadily increases as the temperature decreases from 300 K to 4 K (Fig. 3b). This finding shows that the sample is a semiconductor with the activation energy below 0.05 eV (see supporting information for details, Fig. S5), as averaged from 10 samples. The existence of the semiconducting gap below 0.05 eV suggests that the electrons contributed by potassium to the picene molecules fill the band derived from lowest unoccupied molecular orbitals (LUMO) of picene as predicted by theoretical calculations^21,22^. Nonetheless, the observation of clear semiconducting behavior contradicts the previous theoretical calculation results which predicted metallic behavior^23,24^. Rather, our results match the recent report on weak paramagnetism found in magnetization measurements that suggested the formation of a single ground state in the K_2_picene phase with the estimated band gap of 0.2 eV between the LUMO-derived band and the LUMO + 1-derived band^25^. Based on the results, we believe that K_2_picene is an electrical semiconductor, not a metallic conductor and that the doped electrons are confined to the molecule and do not move freely in the crystal.

### Optical microscopy images and Raman spectra of picene single crystals upon doping

**Figure 4:**
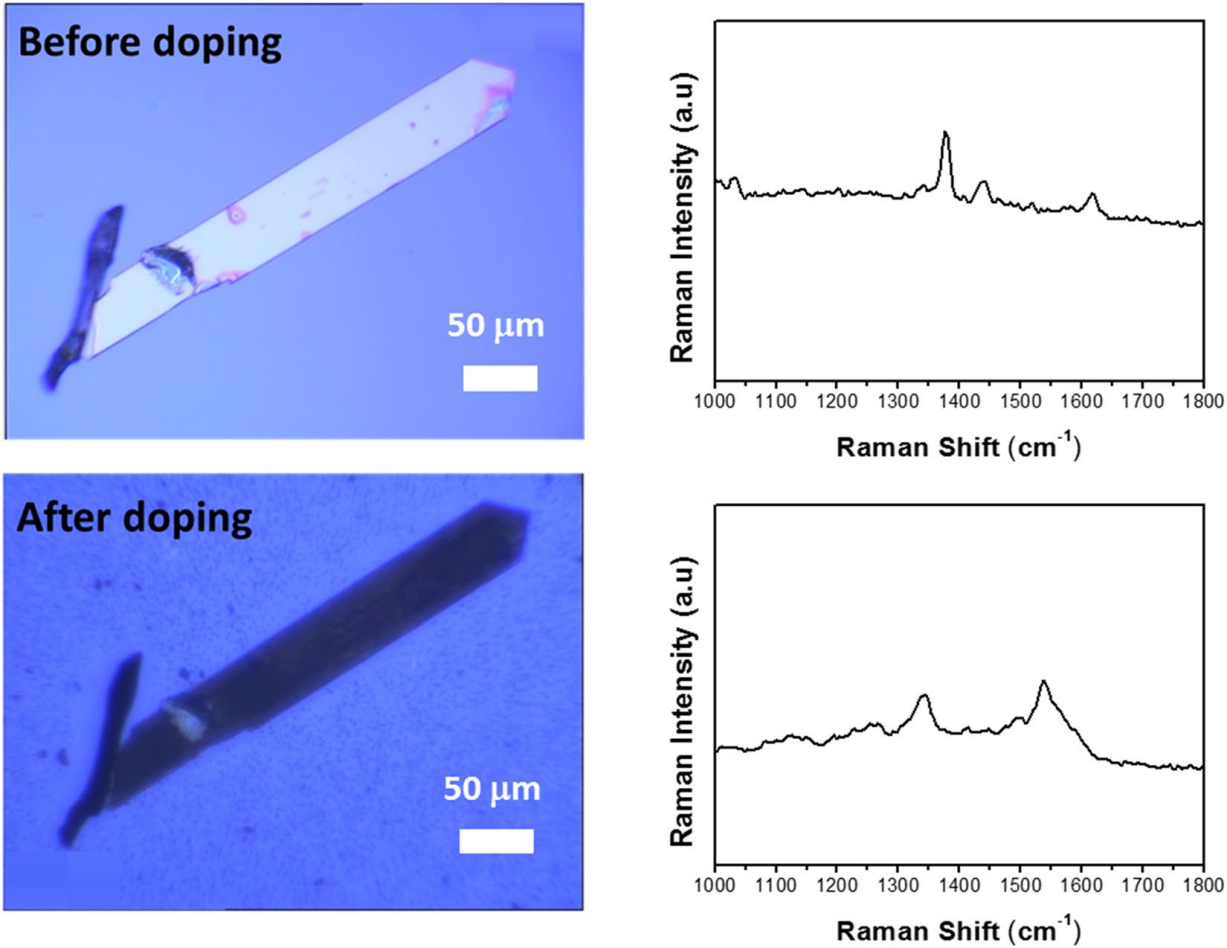
Optical images and Raman spectra of before and after doping process (40 hours) to picene single crystal.

Figure 4 shows the change of the optical image and Raman spectra after the doping process. As the temperature of the substrate was increased to and was kept at 90 °C for 40 hours, alkali metal doping was performed throughout the crystal. The color of the sample was changed from yellow to black, in accordance with the powder doping case^9^. To confirm the doping phase, we used Raman spectroscopy which can identify the state of an anionic molecule. The Raman mode at 1379 cm^−1^ from a doping-free sample is shifted to 1343 cm^−1^ after doping, and this shift is attributed to the emergence of a dianion, which is a signature of K_2_picene^25,26^. In addition, the broadening of Raman peaks in K_2_picene is believed to originate from the strong electron-phonon coupling among the electrons from alkali metal and Raman vibration modes, which is also observed in the doping to C_60_^27^. Based on a set of Raman spectra measured from random positions, we confirmed the uniformity of the peaks throughout the entire crystal, indicating the homogeneity of the picene dianion doping phase (Fig. S7b).

When the doping was performed without secondary thermal activation of the substrate, i.e., conventional doping, the color change of the crystal was observed only at the edges (Fig. S6). Further doping gives rise to more and larger colored spots which propagate into the inner part of the crystal, but complete doping was not observed. In addition, based on the optical microscopy observations, it appears that the diffusion preferentially occurs along the lateral (*ab* plane) direction rather than the vertical (*c* axis) direction because the color change propagates from the edges toward the inner region of the crystal.

However, when the temperature of the crystal was above 120 °C, the picene single-crystal totally turned pale gray, and the Raman spectrum showed peaks corresponding to the dianion phase, but with a broad background (Fig. S7c). This broad background is similar to the previously reported phenomenon of the appearance of some amorphous phase, which means that crystallinity decreases at high temperatures^25^. In addition, at high temperatures (at 160 °C), the structure of the crystal itself collapsed, and the results of the doping reaction did not occur were observed by optical microscopy and Raman analysis (Fig. S8). These results indicate that it is difficult for the crystals to maintain the crystal structure or sustain the expansion of the crystal lattice due to weak van der Waals interactions between the PAH molecules at high temperatures.

### Change of the crystal structure of picene single crystal after doping

We investigated the crystal structure change of the picene single-crystal after doping. The doped picene single-crystal was protected using a Kapton film and sealed into the X-ray diffraction (XRD) sample holder in a glove box. This process effectively avoids the oxidation of the material that degrades the quality of the sample. To check whether the Kapton film affects the XRD patterns, we also examined the XRD pattern of a specimen without a Kapton film (Fig. S9). All peaks from the original picene single-crystal were preserved without significant changes. The results show that the effect of the Kapton tape in the XRD study is negligible.

**Figure 5:**
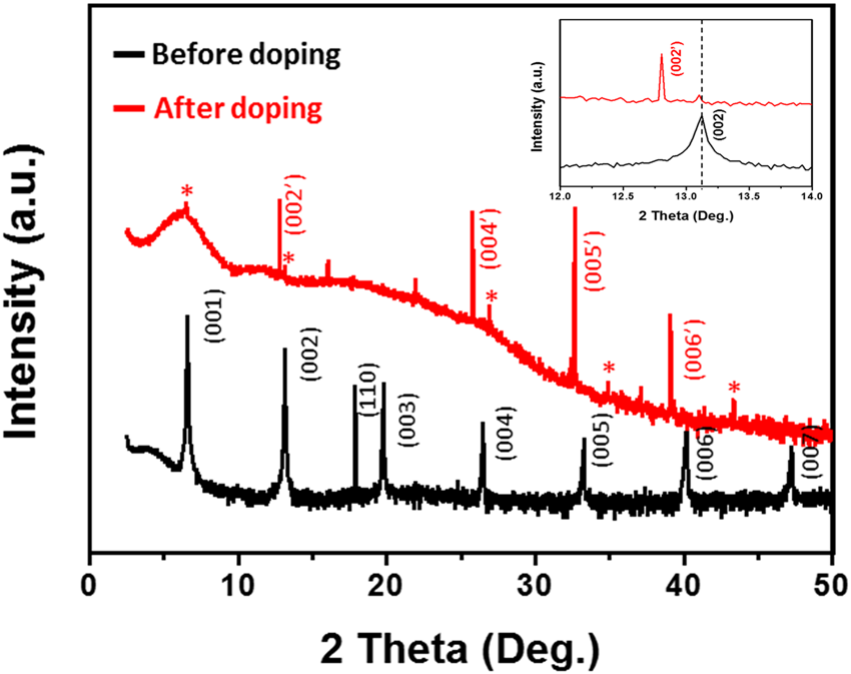
XRD patterns for pristine picene single crystal and K_2_picene crystal after 45 hours of doping process. Inset show magnified XRD patterns around the (002) picene peak.

The X-ray diffraction pattern shows the structural change of the picene crystal induced by the potassium intercalation (Fig. 5). The pristine picene single-crystal has a flat surface with the ab-plane parallel to the substrate (i.e., the (001) planes are parallel to the substrate). Therefore, the [001] diffraction of picene single-crystal is mainly observed, indicating that the sample surface has a preferred orientation.

The XRD pattern shows two different series of peaks after doping that shifted slightly from the preferred orientation position. In Fig. 5, the high-intensity peaks are designated as 00n′, and other peaks with lower intensities are marked by asterisks. We expect that the major intensity peak (00n′) originates from the heavily doped sample because it shows the uniform shift from the original position according to the direction and degree of the shifts predicted by the least square fitting method. However, we could not assign the peaks appearing at 2θ lower than 10 degrees because of the broad background which is attributed to the Kapton tape; an important issue to be addressed is that the peaks related to the pristine picene (00n) peaks were shifted to lower angles, which are fitted to (00n′) planes. This result implies that the d spacing becomes wider between the ab planes and that potassium is intercalated into the c axis (Fig. 5, inset). In addition, the increase in the lattice parameter in the *c* axis does not match the radius of a potassium atom (1.33 Å), implying the possible reorientation of the picene molecules by the interaction between the picene molecules and potassium atoms. Another important issue to address is the absence of KH and KOH peaks in the doped crystal (Fig. S10); these peaks are supposed to appear when the host molecules, i.e., picene in this case, decompose during the doping by hydrogen abstraction, which occurs frequently during the traditional doping process^14^. The presence of such potassium-related species is responsible for the low shielding fraction of the previously reported PAH-based superconductors when these compounds doped using a traditional doping process. Therefore, our method can effectively dope picene single crystals with potassium without the decomposition of the host molecule.

Despite successful doping, superconductivity was not observed through electrical measurement from even more than 10 samples. However, it is noteworthy that the Raman spectra obtained from a few crystals show traces of K_3_picene with a Raman band at 1314 cm^−1^ ^20^ (Fig. S11). Interestingly, the exact stoichiometry in the doped sample, which is three as in A_3_C_60_ (A = alkali metal)^19^, is crucial to induce superconductivity for picene. Therefore, additional electrical characterization is essential to demonstrate the relationship between the K_3_phase and superconducting phenomena, which has remained controversial. However, since the reproducibility has not yet been sufficiently confirmed under certain conditions, no further experiment has been conducted to determine whether or not the K_3_picene phase will exhibit a superconducting state. That is, a K_3_picene single-crystal valuable for the studies of interesting electrical properties including superconductivity can certainly be prepared by our doping method.

## Discussion

Based on the results described above, we propose the following doping mechanism (Fig. 1b). Vaporized potassium adsorbs on the surface, especially near the edge that has more reactive sites of a picene crystal. The key step for the successful doping is the diffusion of the adsorbed potassium atoms into the crystals. We believe that there are two possible driving forces that promote the diffusion of potassium atoms by secondary thermal activation. First, secondary thermal activation may provide additional energy to adsorbed potassium atoms to overcome the energy barrier needed for the diffusion into the well-packed structures of single crystals. The increase of the diffusion rate at higher temperature is a well-known phenomenon, which is also observed in alkali metal intercalation into graphitic structures^28,29^. Another role of secondary thermal activation is to trigger the lattice vibrations or lattice distortions which enable the easier diffusion of the adsorbed potassium atoms into the crystal. Such an “opening” of the host lattice by heat has been reported in glass, where it was found that maintaining the temperature of the host glass materials slightly higher than that of the guest vapor can induce effective diffusion^30^. Uniform doping of the C_60_ single-crystal by potassium has also been achieved by annealing the specimen above 200 °C after the deposition of alkali metal^11^. To verify that the lattice is actually activated by secondary thermal activation, we examined the change in the Raman band at 110 cm^−1^ corresponding to the intermolecular vibration mode of the picene crystal (Fig. S12)^31,32^. *In situ* Raman spectra during doping showed negligible changes in their intensity and energy. That is, thermal activation of the lattice of the target crystal is negligible, and the major effect of the secondary thermal activation would be the provision of kinetic energy to the adsorbed potassium atoms. We note that on the other hand, an excessive heating of the specimen can damage fragile organic crystals for which the intermolecular interactions are much weaker than the bonding interactions of the crystals of inorganic materials or C_60_, and thus, it is important to use a proper temperature in order to apply this method.

## Conclusion

We developed a thermally enhanced diffusion method for alkali metal doping into organic single crystals by applying secondary thermal activation to target crystals. Using this method, we demonstrated the preparation of potassium-doped picene single-crystal with a uniform K_2_picene phase. By contrast, in the absence of the secondary thermal activation of the target crystal, no effective doping was observed. This means that the thermal activation of the crystal during the doping enhances the diffusion of alkali metal atoms. The potassium-doped K_2_picene undergoes a drastic phase transition from an insulator to a semiconductor, with the conductivity above 4.22 × 10^−1^ S/cm. The structure of the doped picene single-crystal was not destroyed, with potassium predominantly intercalated between the molecular layers of picene. One important issue to be addressed during the doping process is the preservation of the molecular structure of picene without the decomposition of the molecule or thermal damage as confirmed by XRD. Although we did not find any metallic or superconducting phase from the doped picene single-crystal at this point, this doping method enables an in-depth investigation of the alkali metal doped molecular semiconductors or superconductors in a single-crystal form.

## Experimental Methods

### Growth of picene single-crystal

All chemicals were used without further purification. The picene single crystals were prepared by the drop-drying method^17^. The saturated precursor solution was prepared as follows: picene (Alpha Aesar, 99%) powder was dissolved in toluene (Alfa Aesar, 98%) and then sonicated for 30 min. The solution was kept at room temperature for 10 min, and then, the saturated solution was filtrated using a syringe filter with the pore size of 0.02 μm (Whatman, Maidstone, Kent, UK) to remove the undissolved picene. A small amount (approximately 20 μL) of the saturated solution was dropped onto a SiO_2_/Si substrate and dried under ambient conditions by allowing the evaporation of toluene. Picene crystals were obtained upon evaporation of the solution. Complete evaporation of the solvent took approximately 15 minutes. The obtained crystals were used for further experiments without any other treatments.

#### Crystallographic Analysis

A colorless picene crystal (0.14 × 0.07 × 0.02 mm^3^) was measured on a ADSC Quantum 210 CCD diffractometer using synchrotron radiation (λ = 0.7000 Å) at the 2D SMC beamline of Pohang Accelerator Laboratory (PAL). The diffraction images were processed using HKL3000. The structures were solved by the direct method (SHELXS-97) and refined by full-matrix least squares calculations on *F*^2^ (SHELXL-2016) using the SHELX-TL program package.

### Fabrication of single-crystal devices

Electrical devices were prepared by transferring the crystal onto a SiO_2_/Si (SiO_2_ = 300 nm) substrate with pre-patterned bottom electrodes. We note that the device has four electrodes so that it can be utilized for four-probe measurement and field-effect transistor measurements. Prior to electrode deposition, the substrate was cleaned by sonicating it in isopropyl alcohol (IPA), acetone, and deionized water for 30 minutes, respectively. The metal electrodes were patterned using a standard photolithography technique. Metallization was performed by deposition of Ti (5 nm) and Pt (25 nm). Picene single-crystal was transferred onto the pre-patterned electrode using a microneedle.

For the measurement of the gate-dependence of picene crystals, a field-effect transistor device was fabricated using CYTOP (80 nm) as the dielectric layer. The CYTOP layer was prepared by spin-coating on a SiO_2_/Si wafer at 3000 rpm for 60 seconds, followed by annealing at 180 °C for 30 min. The channel width and length of the picene single-crystal FET device are 62 μm and 70 μm, respectively. The capacitance per unit area of the dielectric layer are (C_i_) is 8.88 nF/cm^2^.

### Doping of picene single crystals

The doping process was performed using our customized doping system described in Fig. 1a. Briefly, the doping system is composed of two parts. The first contains the alkali metal source (left part in Fig. 1a), and the second is a chamber that contains the sample stage (right part in Fig. 1a). A Cu gasket is used to create a leak-proof connection between two parts. To start the doping process, two parts were assembled in an argon-filled glove box and evacuated by a vacuum pump to below 10^−6^ Torr. Under the dynamic vacuum conditions, the doping process was initiated by heating the alkali metal in the left part which is wrapped with a heating tape. The temperature was measured by a thermocouple attached to the source tube, and was maintained at approximately 140 °C. The evaporated potassium metal vapor is adsorbed onto the surface of picene single crystals on a SiO_2_/Si substrate, which was kept at room temperature. For secondary thermal activation, the sample substrate was heated up to 90 °C using a heating Cu block on which the sample substrate is placed. It should be noted that the connection between the source tube and the sample chamber is overall heated at 90 °C using a heating tape in order to avoid unwanted deposition of potassium metal.

### *In situ* electrical current measurements of picene single-crystal devices during doping

Electrical transport properties during doping were characterized using a semiconductor analyzer (Keithley, 2600). The fabricated devices were mounted on a PCB chip, and thus, the sample can be loaded into the sample chamber and the low temperature measurement system. We monitored the change of the conductance by two-probe measurements during the doping process at the bias voltage of V_DS_ = 1 V, using a semiconductor analyzer.

### Characterization after doping

#### Raman spectroscopy

Raman spectra of crystals were measured using a WITEC Alpha 300 R Raman spectrometer equipped with a 532 nm laser in vacuum conditions (below 10^−6^ Torr). When the electric current fully saturates, the doping was stopped by turning off the source heater. For the Raman measurements, the doping system was detached from the vacuum pump, and loaded into an argon-filled glove box. Then, the doped single crystals were transferred to another chamber with a glass viewport through which laser light and Raman signals can be transmitted while retaining the sample in an inert environment (Fig. S13). The power of the laser was set to 750 μW in order to prevent thermal damage during the measurements, and all Raman measurements were conducted under argon environment.

#### X-ray diffraction measurements

X-ray diffraction (XRD) was carried out at the Pohang Accelerator Laboratory (PAL, Beamline 5D, λ = 1.2401 Å). Note that all XRD data were interpreted using the converted lambda to Cu Kα (λ = 1.54057 Å) value for better comparison with previous reports. The doped single crystals were passivated with a Kapton film and tape in an Ar-filled glove box (H_2_O < 0.1 ppm, O_2_ < 0.1 ppm) to protect the sample from air contact during the XRD measurements. The XRD patterns of KH and KOH were obtained from KH (Alfa Aesar, 30 wt% dispersion in mineral oil) and KOH (Kanto Chemical) powders.

#### *Ex situ* Temperature-dependent resistivity measurements

Picene single crystals after doping were loaded into a homemade cryostat installed in an argon-filled glove box. (Fig. S14) The temperature of the sample was measured with a diode temperature sensor mounted on the substrate, and the resistance was measured using four-probe measurements using a semiconductor analyzer (Keithley 4200-scs model).

## Electronic supplementary material

Supplementary Information

## References

[1] Shirota Y (2000). Organic materials for electronic and optoelectronic devices. J. Mater. Chem..

[2] Sirringhaus H (2014). 25^th^ Anniversary Article: Organic Field-Effect Transistors: The Path Beyond Amorphous Silicon. Adv. Mater..

[3] Hoppe H, Sariciftci NS (2011). Organic solar cells: An overview. J. Mater. Res..

[4] Gunzi S, Yukihiro Y (2007). Development of Conductive Organic Molecular Assemblies: Organic Metals, Superconductors, and Exotic Functional Materials. Bull. Chem. Soc. Jpn..

[5] Takeya J (2007). Very high-mobility organic single-crystal transistors with in-crystal conduction channels. Appl. Phys. Lett..

[6] Wang Z (2014). Metallization and superconductivity of BeH_2_ under high pressure. J. Chem. Phys..

[7] Koike Y, Suematsu H, Higuchi K, Tanuma S (1980). Superconductivity in graphite-alkali metal intercalation compounds. Physica B + C.

[8] Friend RH, Yoffe AD (1987). Electronic properties of intercalation complexes of the transition metal dichalcogenides. Adv. Phys..

[9] Mitsuhashi R (2010). Superconductivity in alkali-metal-doped picene. Nature.

[10] Artioli GA (2015). Superconductivity in Sm-doped [n]phenacenes (n = 3, 4, 5). Chem. Commun..

[11] Xiang XD (1992). Synthesis and electronic transport of single crystal K_3_C_60_. Science.

[12] Palstra TTM, Haddon RC, Hebard AF, Zaanen J (1992). Electronic transport properties of K_3_C_60_ films. Phys. Rev. Lett..

[13] Nicolet MA (1978). Diffusion barriers in thin films. Thin Solid Films.

[14] Heguri S, Kobayashi M, Tanigaki K (2015). Questioning the existence of superconducting potassium doped phases for aromatic hydrocarbons. Phys. Rev. B.

[15] Artioli GA, Malavasi L (2014). Superconductivity in metal-intercalated aromatic hydrocarbons. J. Mater. Chem. C.

[16] De A, Ghosh R, Roychowdhury S, Roychowdhury P (1985). Structural analysis of picene, C_22_H_14_. Acta Crystallogr., Sect. C.

[17] Park C, Park JE, Choi HC (2014). Crystallization-Induced Properties from Morphology-Controlled Organic Crystals. Acc. Chem. Res..

[18] Kawai N (2012). Characteristics of Single Crystal Field-Effect Transistors with a New Type of Aromatic Hydrocarbon, Picene. J. Phys. Chem. C.

[19] Haddon RC (1992). Electronic structure, conductivity and superconductivity of alkali metal doped (C_60_). Acc. Chem. Res..

[20] Kubozono Y (2011). Metal-intercalated aromatic hydrocarbons: a new class of carbon-based superconductors. Phys. Chem. Chem. Phys..

[21] Kusakabe K, Maruyama I, Yamada K (2012). A Theoretical Study Showing K2picene as a Parent Semiconductor for Organic Superconductivity. J. Phys. Soc. Jpn..

[22] Kosugi T, Miyake T, Ishibashi S, Arita R, Aoki H (2011). First-principles structural optimization and electronic structure of the superconductor picene for various potassium doping levels. Phys. Rev. B.

[23] Okazaki H (2010). Electronic structure of pristine and K-doped solid picene: Nonrigid band change and its implication for electron-intramolecular-vibration interaction. Phys. Rev. B.

[24] Naghavi SS, Tosatti E (2014). Crystal structure search and electronic properties of alkali-doped phenanthrene and picene. Phys. Rev. B.

[25] Romero FD (2017). Redox-controlled potassium intercalation into two polyaromatic hydrocarbon solids. Nat. Chem..

[26] Kambe T (2012). Synthesis and physical properties of metal-doped picene solids. Phys. Rev. B.

[27] Dresselhaus, M. S., Pimenta, M. A., Eklund, P. C. & Dresselhaus, G. *Raman Scattering in Materials Science: Raman Scattering in Fullerenes and Related Carbon-Based Materials* (Springer Berlin Heidelberg, Berlin, Heidelberg; 2000).

[28] Zabel H, Magerl A, Rush JJ, Misenheimer ME (1989). Diffusion and melting in two dimensions: A quasielastic neutron scattering study of alkali metals in graphite. Phys. Rev. B.

[29] Wang Z, Ratvik AP, Grande T, Selbach SM (2015). Diffusion of alkali metals in the first stage graphite intercalation compounds by vdW-DFT calculations. RSC Adv..

[30] Kiminori S (2013). Study of Alkali-Metal Vapor Diffusion into Glass Materials. Jpn. J. Appl. Phys..

[31] Protti S (2015). Preparation of (substituted) picenes via solar light-induced Mallory photocyclization. RSC Adv..

[32] Girlando A (2012). Phonon dynamics and electron-phonon coupling in pristine picene. Phys. Chem. Chem. Phys..

