# Supplementary material for: Highly reproducible alkali metal doping system for organic crystals through enhanced diffusion of alkali metal by secondary thermal activation
Source: Sci Rep. 2018 May 16;8:7617. doi: 10.1038/s41598-018-26048-6 (PMC5956060; doi:10.1038/s41598-018-26048-6)
Supplement: Supplementary file 1 — Supplementary Information [file 41598_2018_26048_MOESM1_ESM.docx]

Supplementary Information

**Highly reproducible alkali metal doping system for organic crystals through enhanced diffusion of alkali metal by secondary thermal activation**

Jinho Lee^1,2^†, Chibeom Park^1^†, Intek Song^1,2^, Jin Young Koo^1^, Taekyung Yoon^1,2^ Jun Sung Kim^1,3^ and Hee Cheul Choi^1,2^*

^1^Center for Artificial Low Dimensional Electronic Systems, Institute for Basic Science (IBS), 77 Cheongam-ro, Nam-Gu, Pohang, Korea, 37673

^2^Department of Chemistry, Pohang University of Science and Technology (POSTECH), 77 Cheongam-ro, Nam-Gu, Pohang, Korea, 37673

^3^Department of Physics, Pohang University of Science and Technology (POSTECH), 77 Cheongam-ro, Nam-Gu, Pohang, Korea, 37673

† These authors contributed equally to this work

**To whom correspondence should be addressed. E-mail: choihc@postech.edu*

**Figures**


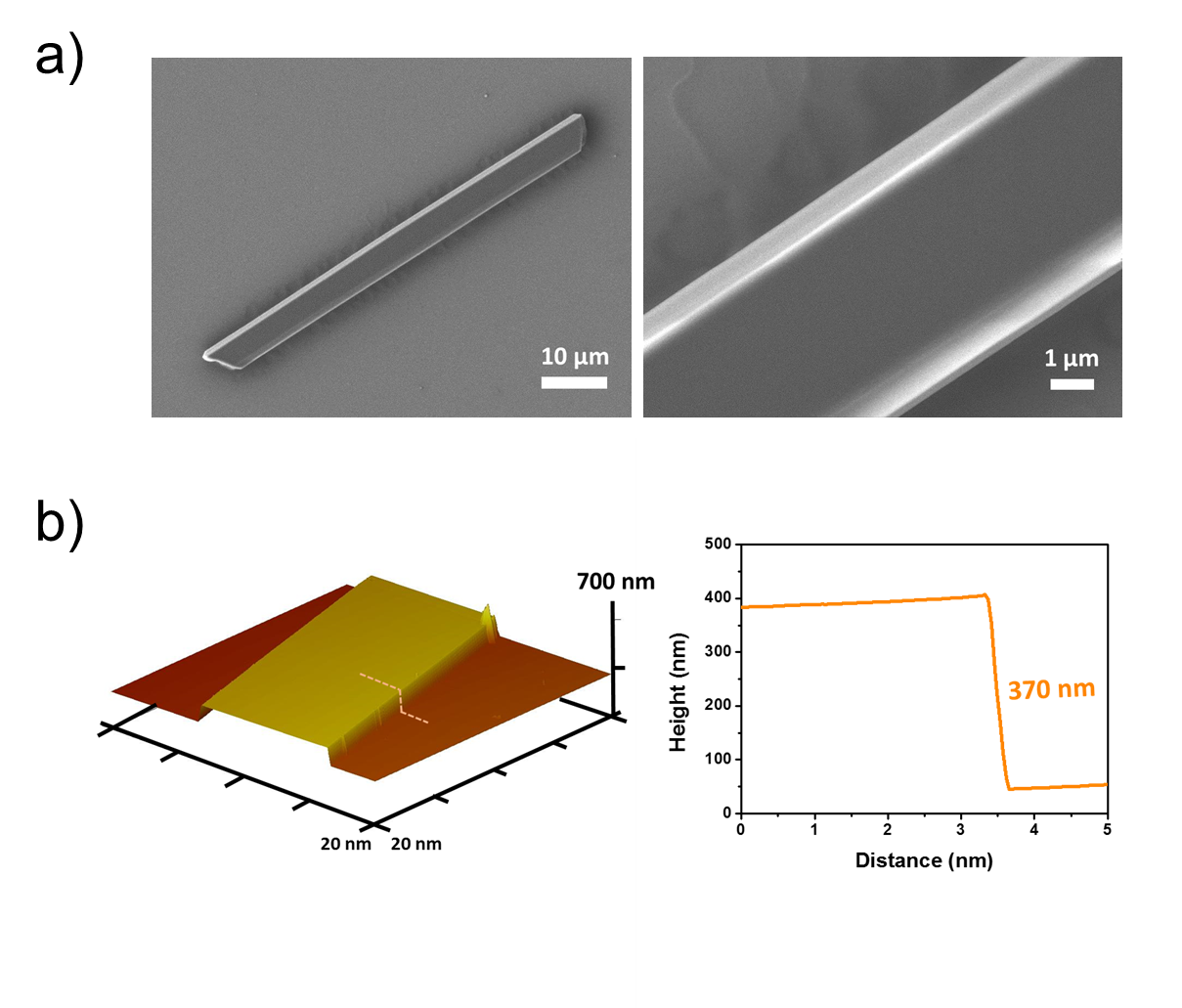


Figure S1. a) a representative SEM image of picene single crystal (left), and a magnified image (right), b) an AFM image of picene single crystal (left) with a height profile (right) along the line indicated in the AFM image.


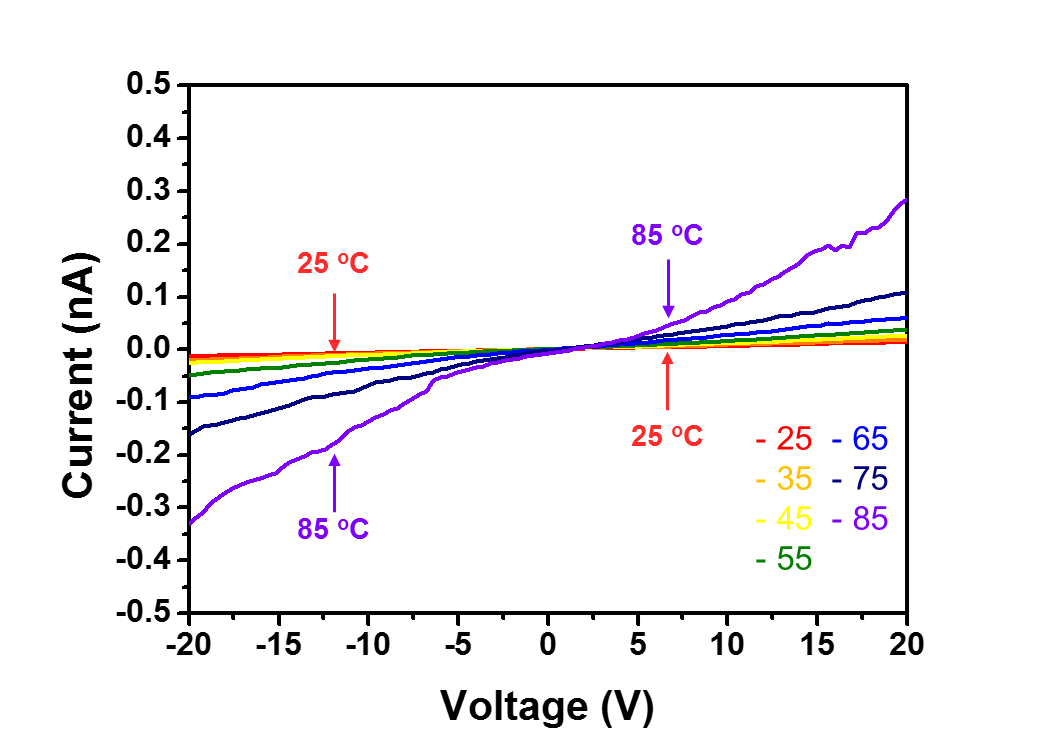


Figure S2. I_DS_-V_DS_ curves measured from a pristine picene single crystal device at temperatures from 25 to 85 °C.


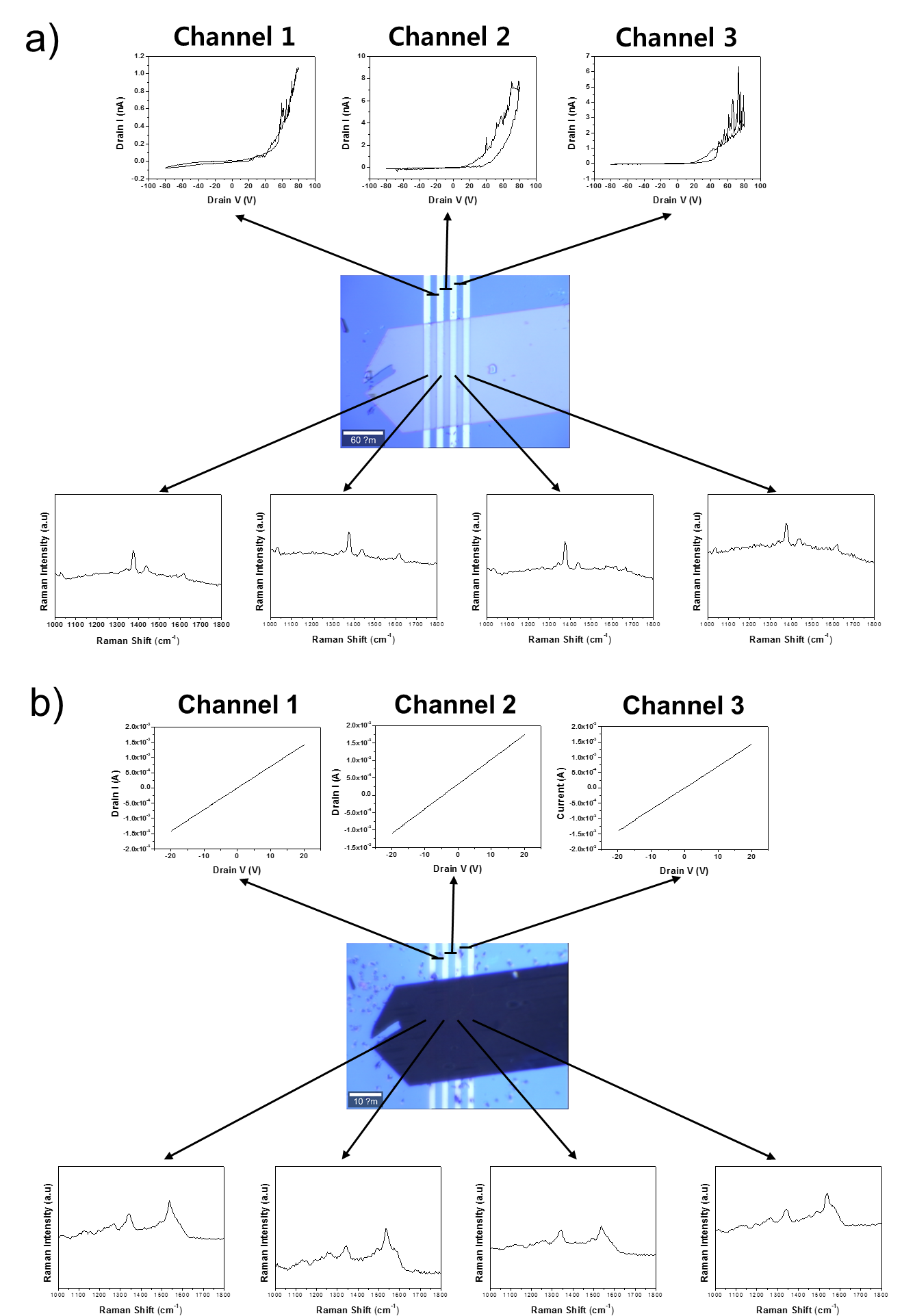


Figure S3. a) I_DS_-V_DS_ characteristic curves (above the optical image) and Raman spectra (under the optical image) taken from each channel region obtained before doping process with an optical microscope image of the undoped picene single crystal. b) I_DS_-V_DS_ characteristic curves (above the optical image) and Raman spectra taken from (under the optical image) each channel region after doping for 45 hrs, with an optical microscope image of the doped picene single crystal.


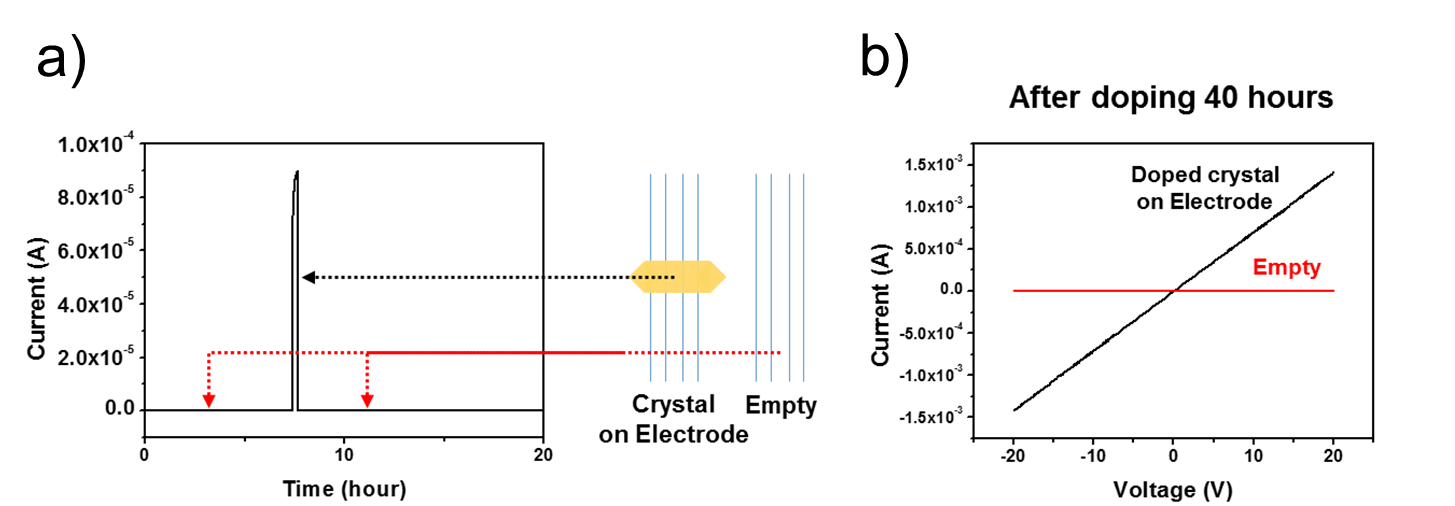


Figure S4 a) Current change during the doping process from picene crystal on electrodes and from empty electrodes. The data from empty electrode is shown together to confirm the sharp current increase in a) owes to the presence of the crystal. b) I_DS_-V_DS_ characteristics after doping for 40 hrs, from doped picene crystal on electrodes (black) and empty electrodes (red).


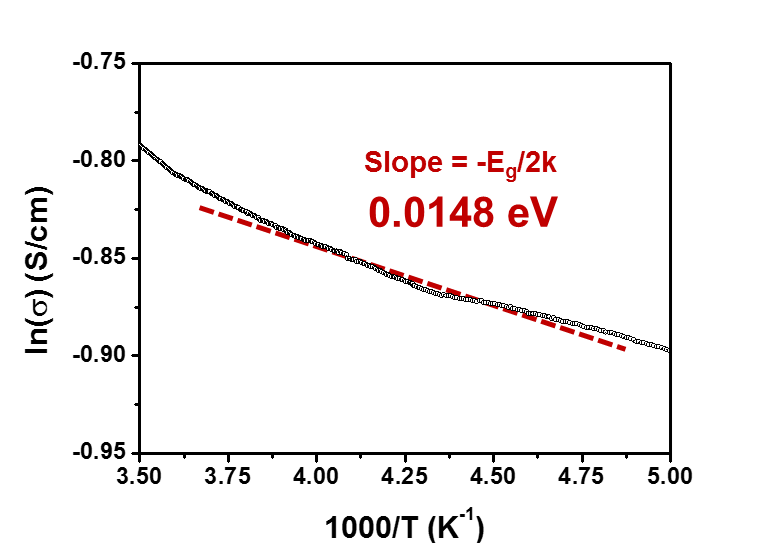


Figure S5. ln(σ) vs 1000/T plot for the K_2_picene single crystal. Temperature linear fit results in a band gap below 0.05 eV using σ ∝σ_0_ exp(-E_g_/2kT)


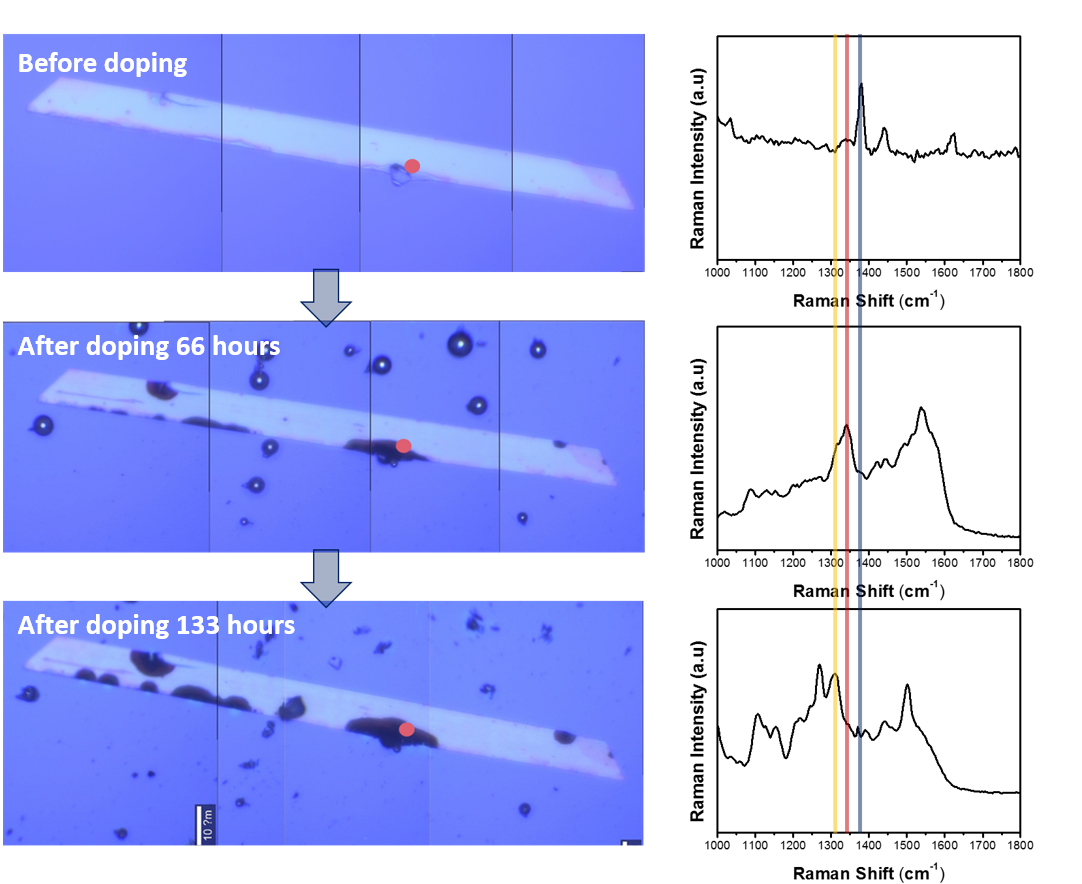


Figure S6. (left column) Optical images of a picene single crystal after doping without secondary thermal activation for indicated doping time (top: pristine, middle: after doping for 66 hrs, bottom: after doping for 133 hrs. (right column) Raman spectra taken from a picene single crystal after doping attempted without the secondary thermal activation. Red dots in each crystal image corresponds to the location from which the Raman spectra were measured.


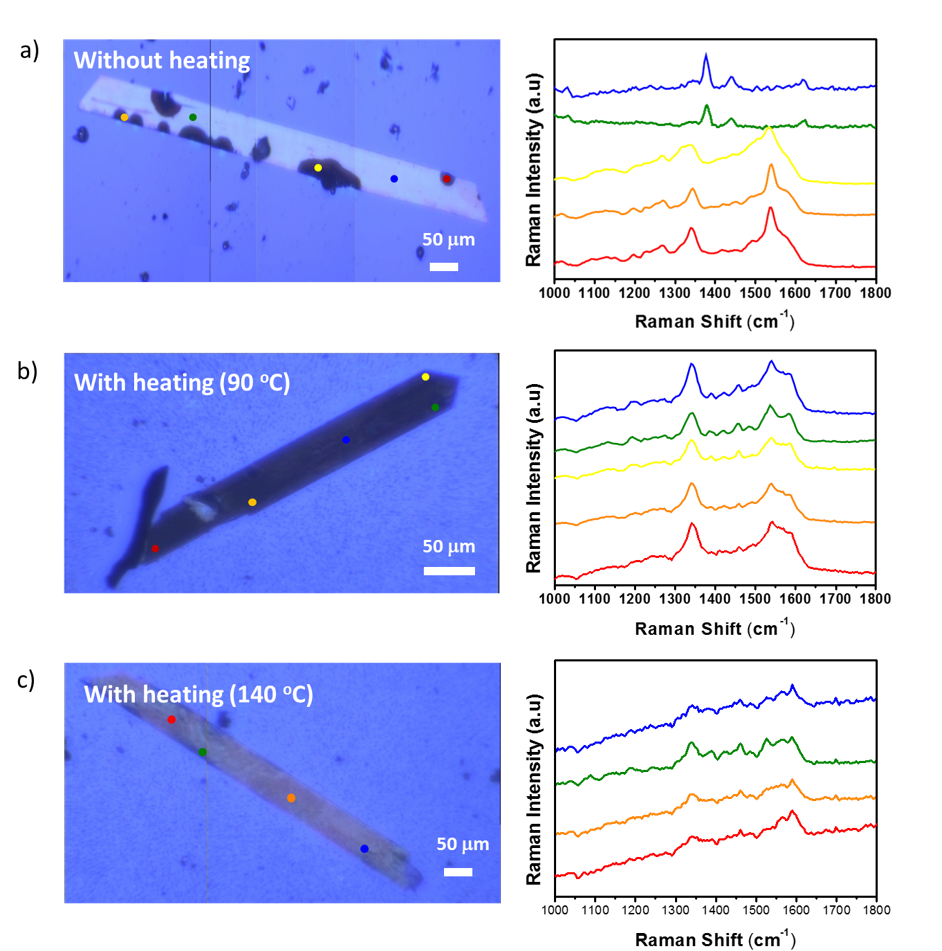


Figure S7. (left column) Optical images of a picene single crystal showing the effect of the secondary thermal activation. a) no secondary thermal activation, b) with the secondary thermal activation by keeping the sample temperature at 90 ^o^C, and c) with the secondary thermal activation by keeping the sample temperature at 140 ^o^C. (right column) Raman spectra obtained from the respective doped picene single crystals in the left column. The color of each spectrum indicates the examined location of colored dots in the corresponding crystal images


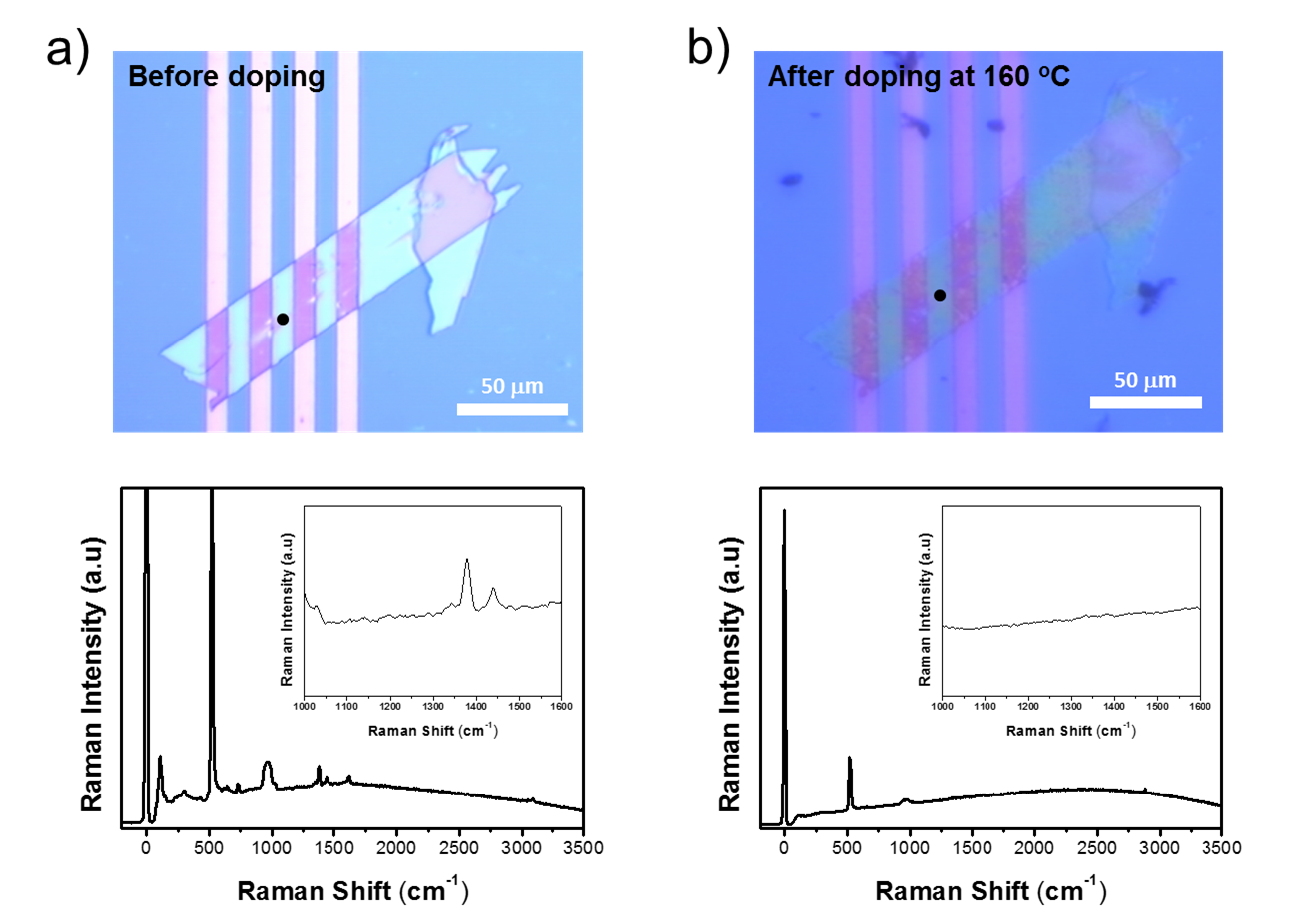


Figure S8. Changes of picene single crystal damaged upon substrate temperature. a) Optical microscope image (top) and Raman spectra (bottom) from pristine picene single crystal on substrate. b) Optical microscope image (top) and Raman spectra (bottom) when doping process was conducted at 160 ^o^C for 40 hours. Black dots in the optical images indicate positions where Raman spectra were measured. Inset spectra are the magnified Raman spectra in the range from 1000 to 1800 cm^-1^.


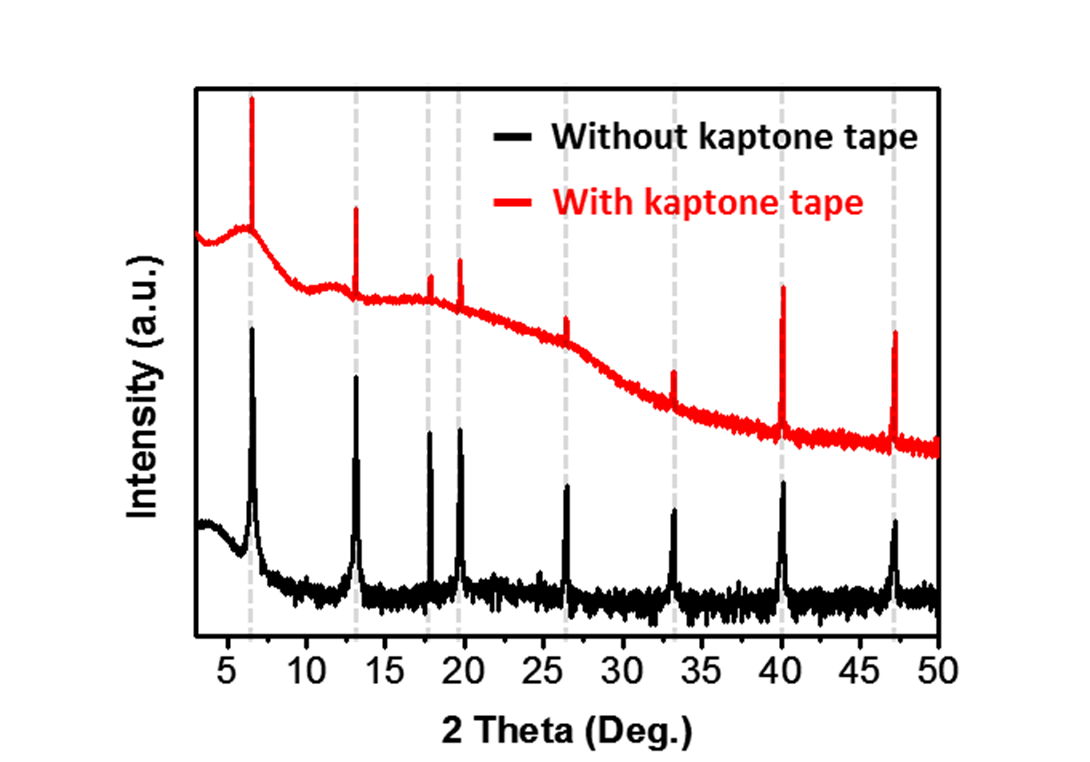


Figure S9. a) XRD patterns of pristine picene single crystal with and without kapton tape.


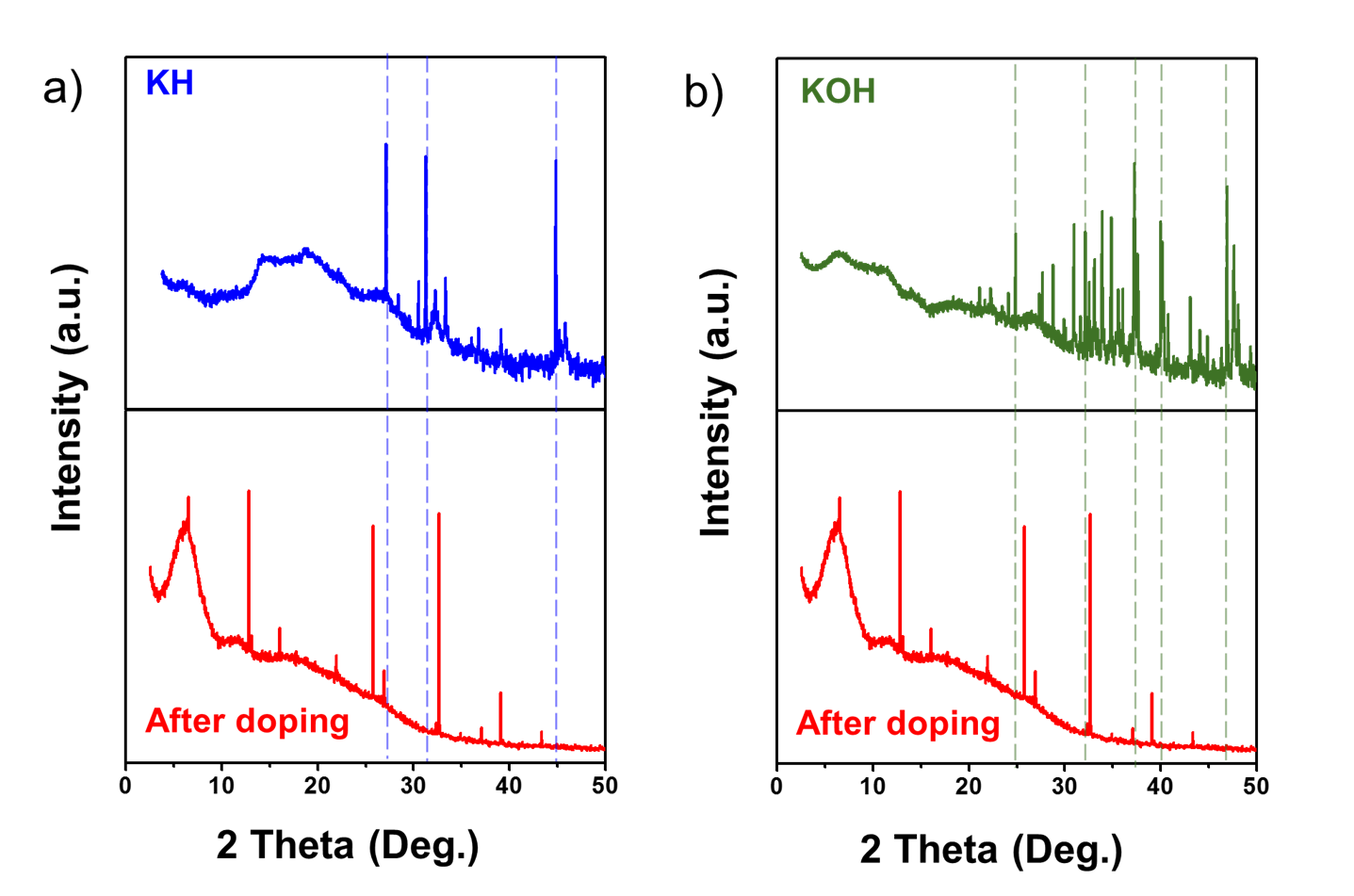


Figure S10. Comparison of XRD patterns of potassium doped picene single crystal after doping for 40 hrs with a) KH powder and with b) KOH powder.


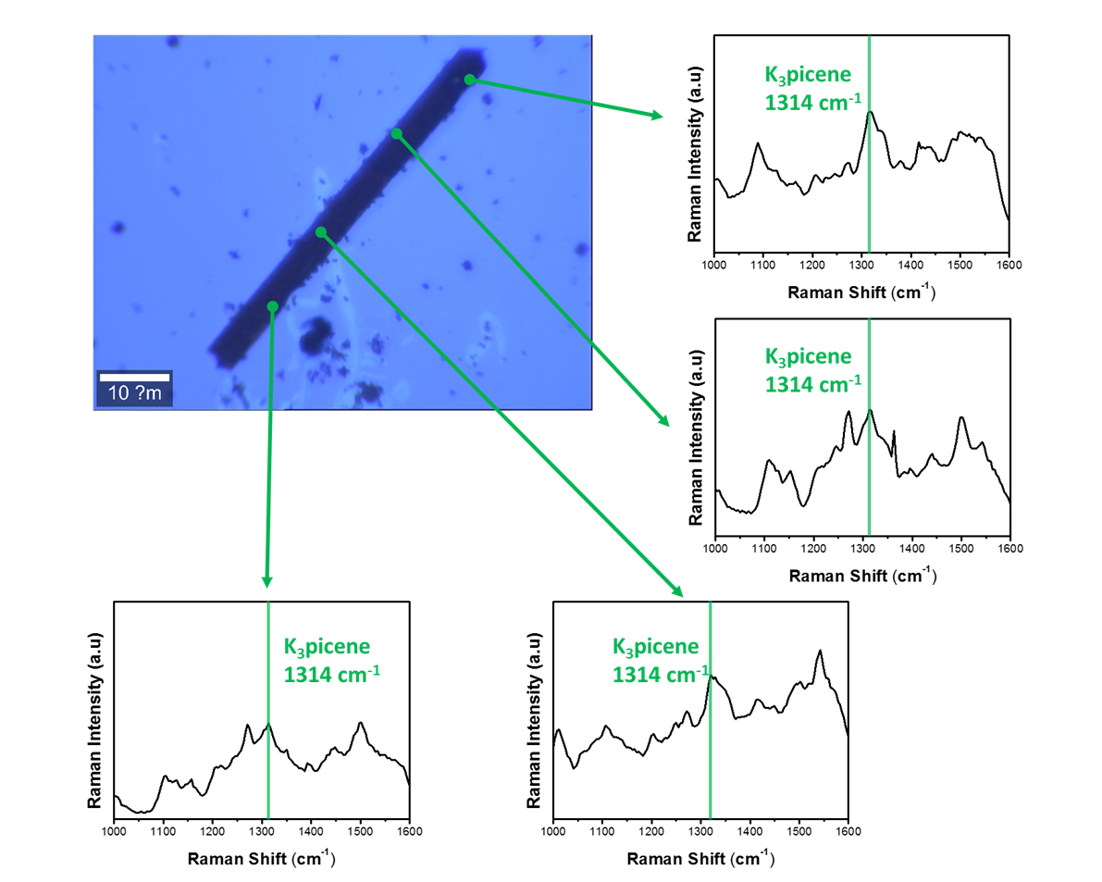


Figure S11. Optical microscope image and Raman spectra of doped picene single crystal having a K_3_picene phase.


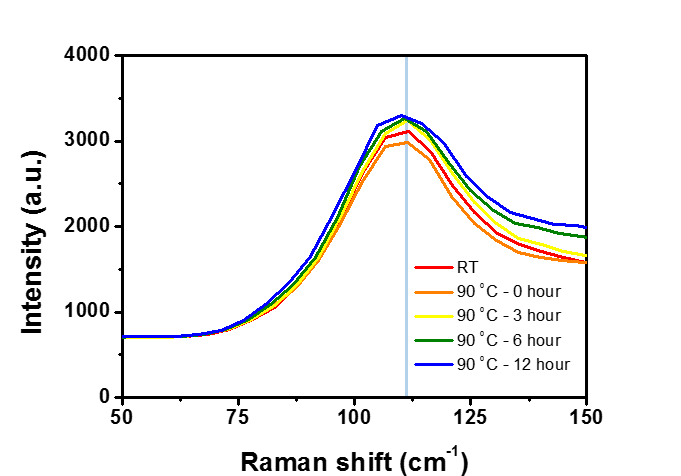


Figure S12. Raman spectra obtained from a picene single crystal treated under secondary thermal activation in the absence of alkali metal to examine the effect of the secondary thermal activation on the structural opening of the crystal upon the activation. The Raman band at 110 cm^-1^ corresponds to the intermolecular vibration mode of picene crystal.


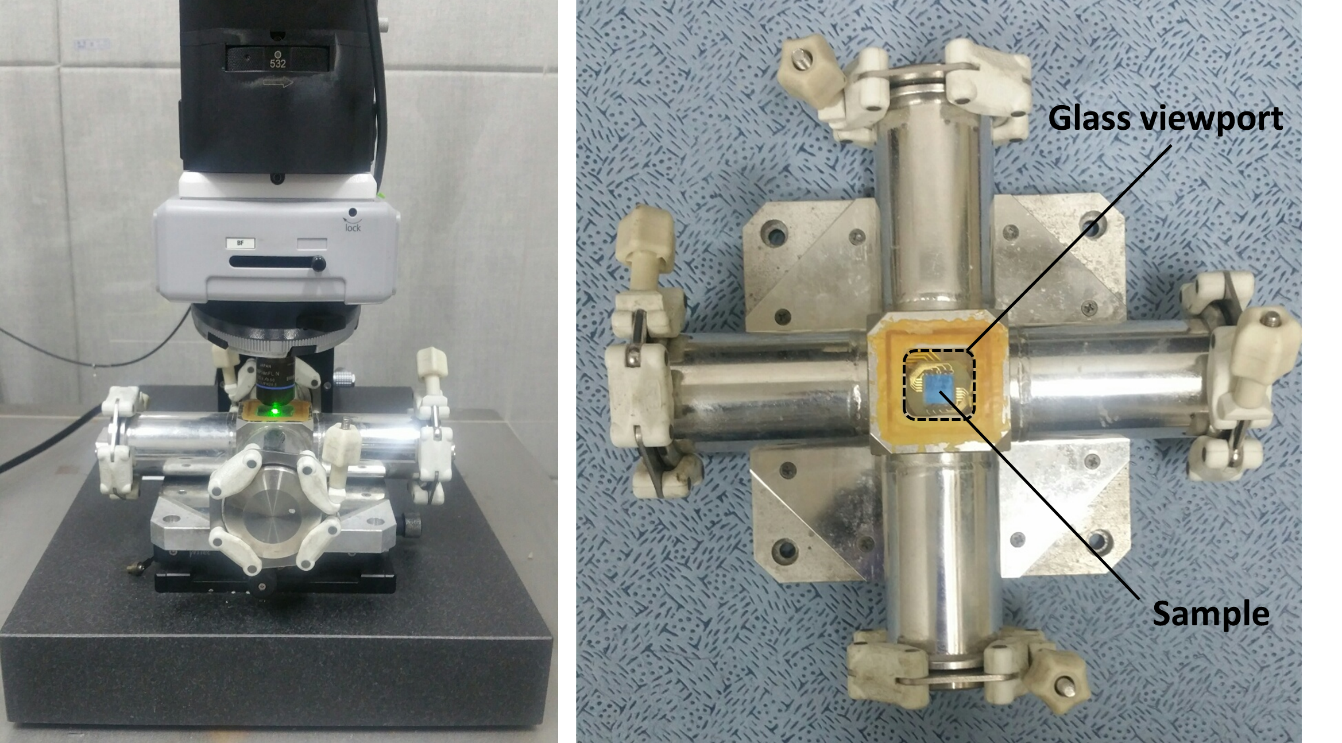


Figure S13. Photograph of experimental setup for Raman spectroscopy.


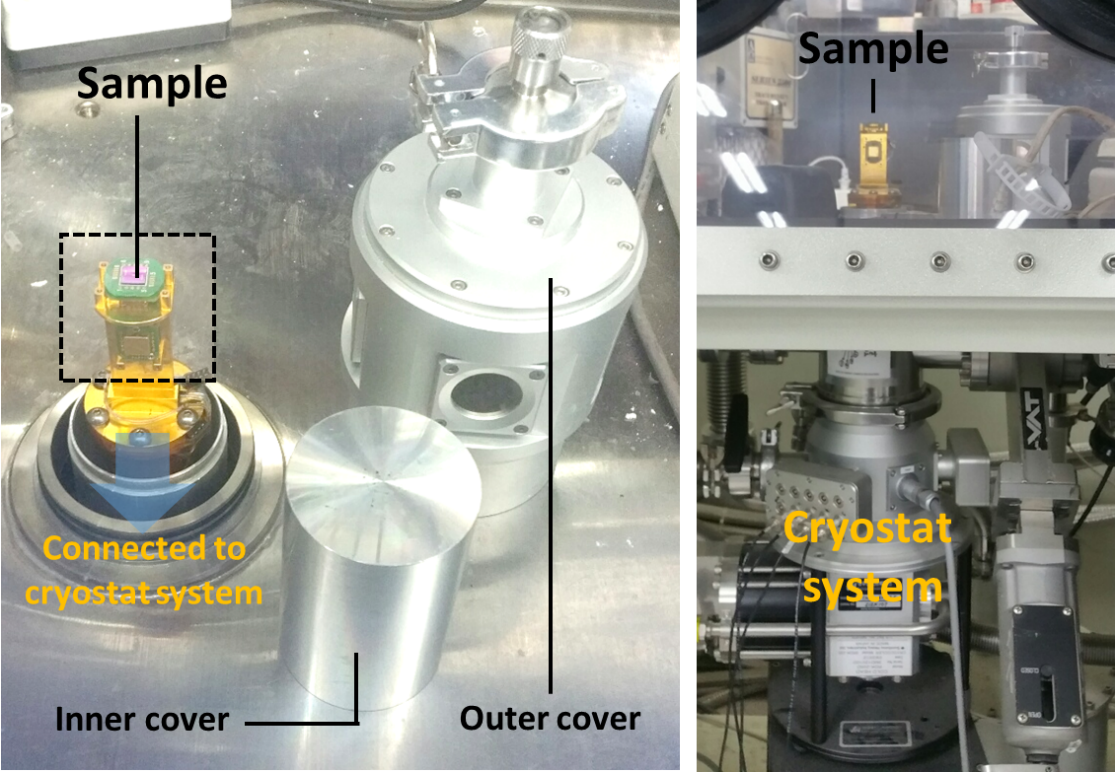


Figure S14. Photograph of cold finger-type cryostat chamber for low temperature electrical measurement installed in a glove box
